# Differences in Walking Pattern during 6-Min Walk Test between Patients with COPD and Healthy Subjects

**DOI:** 10.1371/journal.pone.0037329

**Published:** 2012-05-18

**Authors:** Janneke Annegarn, Martijn A. Spruit, Hans H. C. M. Savelberg, Paul J. B. Willems, Coby van de Bool, Annemie M. W. J. Schols, Emiel F. M. Wouters, Kenneth Meijer

**Affiliations:** 1 Human Movement Science, NUTRIM School for Nutrition, Toxicology and Metabolism Maastricht University Medical Centre, Maastricht, Netherlands; 2 Program Development Centre, CIRO+, Centre of expertise for chronic organ failure, Horn, Netherlands; 3 Respiratory Medicine, NUTRIM School for Nutrition, Toxicology and Metabolism Maastricht University Medical Centre, Maastricht, Netherlands; Leiden University Medical Center, Netherlands

## Abstract

**Background:**

To date, detailed analyses of walking patterns using accelerometers during the 6-min walk test (6MWT) have not been performed in patients with chronic obstructive pulmonary disease (COPD). Therefore, it remains unclear whether and to what extent COPD patients have an altered walking pattern during the 6MWT compared to healthy elderly subjects.

**Methodology/Principal Findings:**

79 COPD patients and 24 healthy elderly subjects performed the 6MWT wearing an accelerometer attached to the trunk. The accelerometer features (walking intensity, cadence, and walking variability) and subject characteristics were assessed and compared between groups. Moreover, associations were sought with 6-min walk distance (6MWD) using multiple ordinary least squares (OLS) regression models. COPD patients walked with a significantly lower walking intensity, lower cadence and increased walking variability compared to healthy subjects. Walking intensity and height were the only two significant determinants of 6MWD in healthy subjects, explaining 85% of the variance in 6MWD. In COPD patients also age, cadence, walking variability measures and their interactions were included were significant determinants of 6MWD (total variance in 6MWD explained: 88%).

**Conclusions/Significance:**

COPD patients have an altered walking pattern during 6MWT compared to healthy subjects. These differences in walking pattern partially explain the lower 6MWD in patients with COPD.

## Introduction

The 6-minute walk test (6MWT) is commonly used to assess functional exercise performance in patients with chronic obstructive pulmonary disease (COPD) [1]. It is a practical, relatively simple test which has gained importance in evaluating the functional status of patients with COPD [2]. Moreover, a poor 6-minute walk distance (6MWD, <350 meters) has prognostic value in patients with COPD [2].

The 6MWD cannot be confidently predicted from conventional descriptors of COPD, such as the Global Initiative for Chronic Obstructive Lung Disease (GOLD) stage or the Medical Research Council (MRC) scale [3]. Therefore, it is necessary to assess functional exercise performance in daily clinical practice in patients with COPD.

**Figure 1:**
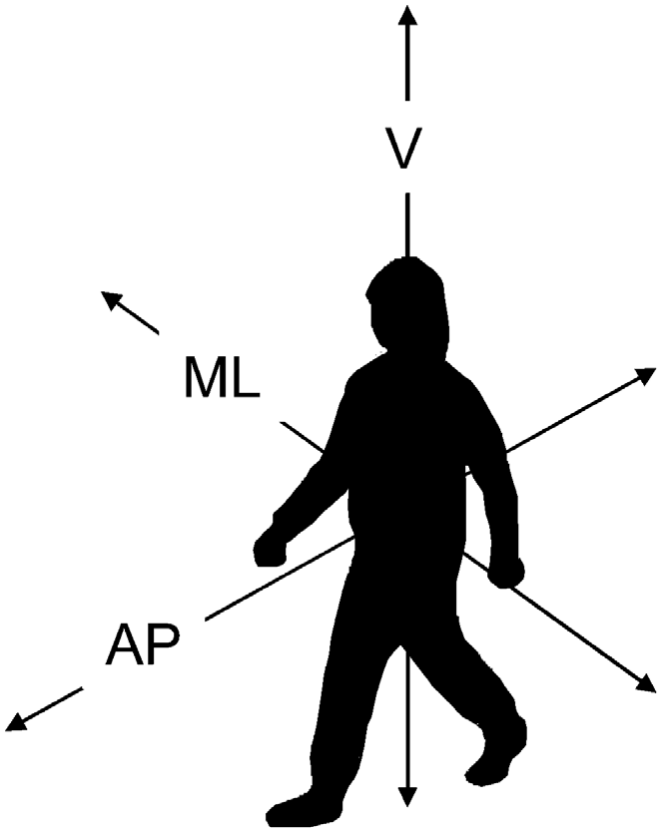
Directions of displacement. AP: Anterior-posterior direction (forward-backward displacement), V: Vertical direction (up-down displacement), ML: medio-lateral direction (left-right displacement)

Walking patterns are generally influenced by the trade-off between the requirements to minimize energetic costs and to maintain stability [4]. Indeed, walking is particularly unstable in the medio-lateral direction (Figure 1). To compensate for balance disturbances during walking, active adjustment of the step width (largely due to through lateral foot placement) is necessary resulting in variability in walking pattern [4]. Then again, to reduce energetic costs of walking, variability in walking pattern needs to be minimized [5]. In COPD patients, different clinical characteristics, such as decreased lower-limb muscle function [6] and a disturbed balance [7], may compromise the ability to balance the energetic and stability requirements posed by walking. Hence, the walking pattern during 6MWT most probably is different between patients with COPD and healthy elderly subjects [8].

Detailed analyses of walking patterns during the 6MWT have not yet been performed in patients with COPD. Yentes et al. recently reported gross walking abnormalities in patients with COPD, such as the presence of a limp or shuffle [9]. These authors used qualitative assessment of gait abnormalities and did not asses the spatiotemporal aspects of walking abnormalities. The latter would enable a direct comparison of walking pattern between patients with COPD and healthy elderly subjects; and an examination of the association between walking pattern, 6MWD and clinical characteristics, like weight, height, the degree of airflow limitation and exercise-induced symptoms of dyspnea and fatigue.

Features derived from tri-axial accelerometers attached to the lower back can be used to measure walking variability. Accelerometers may also allow monitoring walking abnormalities in patients with COPD, as was done before in patients with chronic heart failure [10]. Moreover, routine assessment of exercise performance in a home-based setting in the context of telemedicine seems possible when close associations between accelerometer features and walking distance are found. Therefore, the aim of this study was to determine walking patterns during the 6MWT of COPD patients and healthy elderly subjects. *A priori*, the authors hypothesized that patients with COPD have a different walking pattern during 6MWT compared to healthy elderly subjects, which is related to the reduced 6MWD in COPD independent of the degree of airflow limitation.

## Methods

### Participants

Patients were recruited prospectively during a three-day pre-rehabilitation assessment period at CIRO+, a centre of expertise for chronic organ failure in Horn, the Netherlands [11]. Exclusion criteria were exacerbation-related hospitalization within 4 weeks prior to assessment and the use of a rollator, which is expected to affect the walking pattern [12]. Moreover, patients were excluded from analyses if they were not able to walk at least one 6MWT continuously for six minutes. In all cases non-continuous walks resulted in a worse 6MWD. This is necessary to obtain reliable measures of walking variability [13]. Moreover walking variability cannot be measured over non-walking time.

In total, 93 patients enrolled, of which 14 (15%) stopped during both 6MWT. Therefore, 79 COPD patients were included in the analyses (n = 8 GOLD 1, n = 36 GOLD 2, n = 28 GOLD 3, n = 7 GOLD 4). None of the remaining patients received long-term oxygen therapy (LTOT). All measurements were part of routine baseline assessment for pulmonary rehabilitation [11]. Furthermore, 24 healthy elderly subjects were recruited. Healthy volunteers were recruited amongst healthy subjects who participated in previous trials [14]. None of the healthy subjects used physician-prescribed drugs. The study complied with the Declaration of Helsinki and was approved by the local university's ethics committee (NL30763.068.09). Informed consent was provided by all participants.

### Study protocol

Participants performed two 6MWTs on consecutive days [15]. During both tests an accelerometer (Minimod, McRoberts, The Hague, The Netherlands; size: 8.5×5.0×1.0 cm, weight: 70 g, +/−2G, 100 Hz sampling frequency) was attached to the trunk at the level of the sacrum using an elastic belt to collect raw signalling data. Data obtained during the 6MWT resulting in the highest distance were used for further analyses. Prior to and immediately after each 6MWT participants were asked to report dyspnea and fatigue on a ten-point Borg scale. The best 6-minute walk distance was expressed as a percentage of the predicted values [16].

Post-bronchodilator forced expiratory volume in the first second (FEV1) and forced vital capacity (FVC) were determined using spirometry and reference values were from Quanjer et al. [17]. Moreover, in the COPD patients residual volume (RV) and total lung capacity (TLC) were determined using a whole-body plethysmography to calculate the RV/TLC ratio as a measure of air-trapping. Height and weight were assessed to obtain body mass index (BMI, body weight in kilograms divided by squared height in meters, kg/m^2^). Patients also underwent physical examination and medical history [18], Bio-electrical impedance analysis was used (Bodystat 1500) to determine fat-free mass (FFM) and disease specific equations were used to calculate fat-free mass index (FFMI) [19].

### Data Analysis

For data analyses of the accelerometer signals 5 seconds at the beginning and end of the test were excluded to be sure that possible group differences in walking pattern were not due to start and/or stop of the 6MWT. Dedicated software written in Matlab(c) was used to analyse the remaining 350 seconds of raw acceleration data. The software included algorithms to calculate the walking intensity, spatio-temporal aspects of gait and medio-lateral stability. Walking intensity was calculated from the integral of the modulus accelerometer output [20]. For this purpose, accelerometer output was low-pass filtered with a fourth-order Butterworth filter (20 Hz). The absolute value of the residual signal was taken to rectify the signal. After this process, the area under the curve over the complete measurement was calculated by integrating the signal over a period of 350 seconds. This integration was done separately for all three measurement directions (i.e. frontal, horizontal and sagittal plane). The integral of the modulus accelerometer output was then obtained by summation of these values [20]. Onsets of support phases were determined from forward accelerations as described by Zijlstra et al. [21]. During the transition from single to double support (i.e. after contra-lateral foot contact), the forward acceleration of the lower trunk changes sign from positive to negative. The peak forward acceleration preceding the change of sign coincides with the instant of foot contact. The acceleration peak preceding a change of sign (from positive to negative) was taken as the instant of a left or right foot contact. Consequently the strides ( = 2 steps) were identified. The cadence (strides/min) was calculated from the mean stride times. The inter-stride trunk acceleration variability was calculated using an unbiased autocorrelation coefficient procedure [22], which previously has been used to identify walking patterns of frail and fatigued elderly [5], [22]. The autocorrelation function estimates how a time series is correlated with itself over different time lags. For a time series of trunk accelerations during walking, autocorrelation coefficients can thus be produced to quantify the peak values at the first and second dominant period, representing phase shifts equal to one step and one stride, respectively. Variability, as measured by the autocorrelation coefficients were calculated for the anterior-posterior, vertical and medio-lateral direction [23]. A higher autocorrelation coefficient indicates lower between-stride time variability (range: 0 to 100%).

### Sample Size and Power

Sample size and power sample size calculations are based on outcomes of Moe-Nilsson et al. using the interstride trunk acceleration variability of fit and frail older adults [22]. Twenty-two participants in each group would provide 80% power at alpha 0.05 (two-tailed) to detect differences between COPD patients and healthy subject of 8% with a standard deviation of 10%. To cover a larger spectrum of COPD severity by having enough patients in disease stages 1/2 and 3/4, 93 COPD patients enrolled the study.

### Statistical Analysis

Statistical analysis was done using SPSS software (version 15.0, SPSS Inc.). Data are reported as mean ± standard deviation (SD) or percentages, as appropriate. GOLD stages 1 and 2, and GOLD stages 3 and 4 were combined for further analyses due to the small number of GOLD stage 1 (n = 8) and GOLD stage 4 patients (n = 7). The comparisons were conducted with 1-way analysis of variance or chi-square tests as appropriate. Accelerometer features (walking intensity, cadence, variability in anterior-posterior, vertical and medio-lateral direction), subject characteristics (gender, age, height, weight and FEV1) and perceived dyspnea and fatigue (before and after the best 6MWT) were tested in their association with the 6MWD via multiple ordinary least squares (OLS) regression models per group. Previously, different patterns were found for the variability in medio-lateral direction *versus* anterior-posterior and vertical directions between frail or fatigued persons and fit persons [5], [22]. Therefore the interactions between variability for the different directions were also tested. Multicollinearity tests were carried out variables were retained in the model if the variance inflation factor was smaller than 5.0. A top-down procedure was handled for the selection of the final model variables. Accelerometer features probably rely, at least in part, on the walking speed. Therefore, *a posteriori* walking variability measures were compared in a subset of healthy subjects and COPD patients who had on average a comparable walking distance (range 6MWD: 560 m–640 m). Differences in walking patterns between the best and worst 6MWT in patients with COPD are described in the Text S1, table S1 and table S2. *A priori*, results were considered statistically significant when p-value was ≤0.05.

## Results

### Characteristics

Healthy subjects and COPD patients had a similar gender distribution, age and BMI (table 1). As expected, GOLD stage 3/4 patients had the worst 6MWD, also after correction for confounding variables, like height, weight, age and gender [16]. COPD patients experienced more fatigue and dyspnea during the 6MWT compared to healthy elderly subjects.

**Table 1:** Patients' characteristics.

	Healthy	All COPD	GOLD 1/2	GOLD 3/4
	(n = 24)	(n = 79)	(n = 44)	(n = 35)
Men (%)	62.5	59.5	59.1	60.0
Age (yrs)	63.7 (5.9)	64.3 (8.9)	64.9 (8.5)	63.5 (9.5)
Height (m)	1.73 (0.07)	1.67 (0.09)*	1.67 (0.10)*	1.66 (0.06)*
Weight (kg)	79.5 (13.2)	69.0 (15.0)*	70.1 (16.3)*	67.5 (13.2)*
BMI (kg/m^2^)	25.8 (3.8)	24.7 (4.5)	25.0 (4.6)	24.3 (4.3)
Tiffeneau index (%)	77.0 (4.1)	40.7 (11.9)*	47.6 (9.3)*	31.9 (8.4)* #
FEV1 (%pred)	124.9 (21.0)	53.5 (18.7)*	66.6 (14.0)*	37.0 (7.2)* #
RV/TLC ratio (%)	-	50.5 (10.0)	45.6 (8.9)	56.8 (7.5)#
6MWD (m)	672 (85)	494 (96)*	528 (95)*	451 (79)* #
6MWD (% pred)	102.3 (11.6)	77.6 (13.5)*	83.4 (12.0)*	70.3 (11.7)* #
Baseline dyspnea (points)	0.31 (0.51)	1.64 (1.22)*	1.34 (1.19)*	2.01 (1.18)* #
Δ Dyspnea (points)	1.33 (1.33)	2.74 (1.97)*	2.77 (2.04)*	2.70 (1.91)*
Baseline fatigue (points)	0.50 (0.81)	1. 52 (1.48)*	1.41 (1.53)*	1.66 (1.42)*
Δ Fatigue (points)	1.13 (1.27)	2.25 (1.76)*	2.22 (1.52)*	2.30 (2.04)*
FFM (kg)	-	46.5 (8.8)	47.3 (9.1)	45.6 (8.5)
FFMi (kg/m^2^)	-	16.6 (2.2)	16.8 (2.1)	16.3 (2.3)

Value expressed as mean ± standard deviation (SD).

Abbreviations: BMI: body mass index, FEV1: forced expiratory volume in the first second, RV: residual volume,TLC: total lung capacity, 6MWD: 6-min walk distance, FFM: fat-free mass, FFMi: fat-free mass index.

* : significantly different from healthy subjects. (p<0.05).

# : significantly different from GOLD stage 1/2. (p<0.05).

### Accelerometer features

Accelerometer features showed that COPD patients walked at a significantly lower intensity and a lower cadence (table 2). Differences in intensity and cadence were also found between patients in GOLD stages 3/4 and GOLD stages 1/2. Moreover significantly increased variability (as measured by the lower autocorrelation coefficients) was found for the medio-lateral acceleration in the COPD group compared to healthy controls.

**Table 2:** Accelerometer features.

	Healthy	COPD	GOLD 1/2	GOLD 3/4
	(n = 24)	(n = 79)	(n = 44)	(n = 35)
Walking intensity (counts/min)	14054 (3198)	8658 (2971)*	9892 (3214)*	7106 (1654)* #
Cadence (strides/min)	66 (4)	57 (6)*	59 (5)*	55 (5)* #
AC-AP (%)	81.9 (10.4)	79.0 (10.7)	79.4 (9.7)	78.5 (12.0)
AC-V (%)	87.3 (6.9)	84.2 (10.2)	85.6 (6.8)	82.5 (13.3)
AC-ML (%)	73.7 (12.5)	63.2 (14.0)*	64.4 (12.4)*	61.6 (15.9)*

Value expressed as mean ± standard deviation (SD).

Abbreviations: AC-AP: autocorrelation coefficient in anterior-posterior direction, AC-V: autocorrelation coefficient in vertical direction, AC-ML: autocorrelation coefficient in medio-lateral direction.

* : significantly different from healthy subjects. (p<0.05).

# : significantly different compared to GOLD 1/2. (p<0.05).

### A *posteriori* analysis

Healthy subjects (n = 8) and COPD patients (n = 14) with a 6MWD range between 560 m and 640 m did not differ significantly in 6MWD (healthy: 595±13 m, COPD: 596±20 m, p = 0.869), walking intensity (healthy:11782±1083 a.u., COPD: 11501±2013 a.u., p = 0.720) and cadence (healthy: 64±3 strides/min, COPD: 61±4 strides/min, p = 0.107). Nevertheless, the COPD patients maintained to have a significantly lower autocorrelation coefficient in the medio-lateral direction compared to the healthy control subjects (healthy: 81±7%, COPD: 67±12%, p = 0.003).

### Determinants of 6MWD

**Figure 2:**
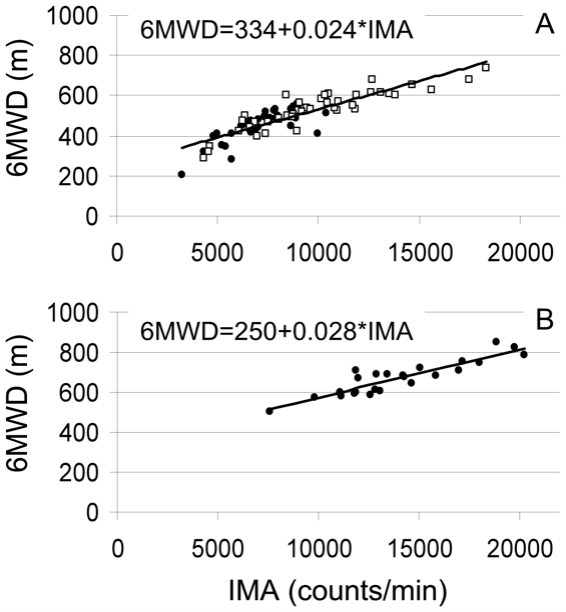
Relation between the accelerometer output walking intensity per min and the 6MWD. A: GOLD stages 1 and 2 with open squares and GOLD stages 3 and 4 with closed circles and B: Healthy subjects

The intensity parameter walking intensity correlated most strongly with the 6MWD in healthy subjects (r = 0.902, p<0.001) and COPD patients (r = 0.872, p<0.001) (figure 2). The correlation between 6MWD and FEV1 in COPD (r = 0.452, p<0.001) and between walking intensity and FEV1 in COPD (r = 0.495, p<0.001) were both significant. No significant correlations between these variables were found in healthy subjects.

The results of the OLS regression models used to test associations between subject characteristics, accelerometer features and 6MWD for healthy subjects and COPD patients (all GOLD stages) are summarised in table 3. The model variables explained 85% and 88% of the variability in 6MWD in healthy subjects and COPD patients, respectively. walking intensity and height were the only two significant determinants of 6MWD in healthy elderly subjects. In patients with COPD also age, cadence, walking variability measures and their interactions were included.

**Table 3:** Model variables influencing the 6MWD – Linear Regression.

	Healthy	COPD
	(n = 24)	(n = 79)
Height (m)	242.6 [54.4–430.3]	244.8 [132.4–357.2]
Age (y)	NS	−1.03 [−2.02–−0.04]
Walking intensity (counts/min)	0.023 [0.018–0.028]	0.019 [0.016–0.023]
Cadence (strides/min)	NS	3.01 [0.81–5.20]
AC-AP (%)	NS	15.15 [5.30–24.99]
AC-V (%)	NS	10.19 [4.28–16.11]
AC-ML (%)	NS	−10.84 [−17.09–−4.60]
AC-AP*AC-V (interaction)	NS	−0.18 [−0.30–−0.06]
AC-V*AC-ML (interaction)	NS	0.12 [0.05–0.19]
Model Constant	−70.82 [−391.68–250.04]	−991.1 [−1491.9–−490.26]
Adjusted Model R^2^	0.85	0.88

Value expressed as Beta's (ß) ±95% confidence interval [CI].

Abbreviations: AC-AP: autocorrelation coefficient in anterior-posterior direction, AC-V: autocorrelation coefficient in vertical direction, AC-ML: autocorrelation coefficient in medio-lateral direction.

Note: effects of the following variables were also tested, but no statistical significance was detected: Gender, weight, FEV1, perceived dyspnea and fatigue (before – after the 6MWT), interaction between the autocorrelation coefficient in anterior-posterior and medio-lateral direction.

## Discussion

The present study provides the first comprehensive evaluation of qualitative and quantitative measures of the walking pattern during 6MWT in patients with moderate to very severe COPD. It extends previous work on the 6MWT by providing detailed information on walking variability. On average, COPD patients walk with a lower intensity, a lower cadence and show a higher medio-lateral variability during 6MWT in comparison with healthy elderly subjects. The difference in medio-lateral variability remained even if the walking speed was similar. Moreover, walking variability was associated with functional exercise capacity in COPD patients, but not in healthy controls. These results indicate an altered walking pattern in COPD patients.

Increased variability in the medio-lateral direction (largely due to through lateral foot placement) is an active control strategy to compensate for balance disturbances in order to maintain stability in the anterior-posterior direction (the direction of propulsion). In the present study, walking variability was higher in the medio-lateral direction in COPD patients compared to the control group (table 2), suggesting larger balance disturbances during the 6MWT in the patients. This may at least in part contribute to the relatively high energetic costs of a 6MWT in patients with COPD [24]. Moreover, this may also explain partially why patients with COPD experience abnormalities with day-to-day walking [25], including falls [26]. Indeed similar deviations in walking patterns were previous observed in frail elderly who fell at least once during the last year or who used walking aid [22].

A high positive association was found between the intensity parameter walking intensity and 6MWD in both COPD patients and healthy controls (figure 2). Previously, similar findings were reported in patients with chronic heart failure [10], [27]. These high associations create future possibilities for routine assessment of exercise performance of patients in a home-based setting in the context of telemedicine. Moreover, multiple accelerometer features and subject characteristics explained 88% of the variability in 6MWD in the patients with COPD. Next to familiar determinants of the 6MWD in COPD (i.e., height and age), also a higher walking intensity, a higher cadence, lower variability in anterior-posterior and vertical directions, and a higher variability in medio-lateral direction were significantly associated with a higher 6MWD (table 3). Moreover, interactions between variability in anterior-posterior and vertical direction and between vertical en medio-lateral direction were found. The degree of airflow limitation (e.g., FEV1) did not significantly explain the variance in 6MWD in a multiple model.

Previously, the cadence and the walking intensity have been studied in patients' home-environments to evaluate daily performance in COPD patients or to study the effects of pulmonary rehabilitation [28]. The present study shows that walking variability is also a clinically relevant variable in COPD patients as it significantly contributes to the prediction of 6MWD. This is an important finding as lower 6MWD has been related to more exacerbation-related hospitalizations and higher mortality rates in patients with COPD [2].

The design of this study was cross-sectional. Future investigations using the current methodology should include repeated measures to investigate the effect of a comprehensive pulmonary rehabilitation program on walking pattern in patients with COPD. Indeed, faster walking may be more stable than slow walking [29]. On the other hand, this study showed that also the COPD patients with the same walking speed as healthy subjects have an increased variability in the medio-lateral direction. This strongly suggests that the differences found in walking variability between COPD and healthy cannot be attributed only to differences in walking speed. It is therefore more likely that in COPD patients walking stability is influenced by dyspnea [30], altered breathing dynamics [31], reduced arm swing, [32], lower muscle strength and/or coordination [5], [6], disturbed balance [7], [33], or a combination thereof. Accelerometers may be helpful to evaluate 6-min walking patterns as an index of treatment outcome. Moreover current findings should be reproduced in the patients own environment.

Participants who stopped during both 6MWT were excluded from this study, which was necessary to obtain reliable measures of walking variability [13]. As a result, the 6MWD of the COPD patients was higher compared to previous studies [15], [34]. Then again, the current mean 6MWD of 494 m is well within the range in 6WMD as observed in the ECLIPSE study [3]. The Modified Medical Research Council (MMRC) Dyspnea Scale was not assessed, therefore it cannot be excluded that dyspnea may have contributed to the 6MWD in patients with COPD [3]. The present results are hypothesis-generating rather than definitive. Future studies are warranted to corroborate the present findings and to explain why patients with COPD have a different walking pattern compared to healthy elderly subjects. This may be due to a variety of factors [5], [6], [7]. Moreover, the current findings generate a clear rationale to study in detail walking patterns using tri-dimensional analyses, including electromyographic activity of lower-limb muscles [35].

In conclusion, patients with COPD have a different walking pattern during 6MWT compared to healthy elderly subjects, as objectified by using accelerometer signals. In addition to walking intensity, cadence and walking variability are important variables associated with 6MWD in patients with COPD. These differences in walking pattern partially explain the reduction in 6MWD in patients with COPD.

## Supporting Information

Text S1 **Intra-individual differences between best and worst 6-min walk tests.**
(DOCX)

Table S1 **Patients' characteristics for all COPD patients and a subgroup of COPD patients with a 2nd test.**
(DOCX)

Table S2 **Accelerometer features.**
(DOCX)
