# Supplementary material for: Differences in Walking Pattern during 6-Min Walk Test between Patients with COPD and Healthy Subjects
Source: PLoS One. 2012 May 18;7(5):e37329. doi: 10.1371/journal.pone.0037329 (PMC3356256; doi:10.1371/journal.pone.0037329)
Supplement: Text S1 — Intra-individual differences between best and worst 6-min walk tests. (DOCX) [file pone.0037329.s001.docx]

# Online supplement text S0

**Intra-individual differences between best and worst 6-min walk tests**

Methods

From 49 patients out of 79 patients accelerometer recordings were obtained from continuous walking during two 6-min walk tests. Data from this subgroup of patients were used to test the intra-individual differences between best and worst 6-min walk tests using a paired samples t-test. Patient characteristics of this subgroup are similar to the characteristics of all participants included in the study and described in table S1.

Results

COPD patients walked during the best test significantly more distance (32m). Moreover COPD patients walked during the best test with an increased intensity, with a higher cadence and with decreased variability as measured by the autocorrelation coefficient in the vertical and medio-lateral directions (table S2).

Conclusion

The results show clear intra-individual differences between best and worst 6-min walk. The accelerometer features are sensitive to these small differences in 6-minute walk distance.
