# Supplementary material for: Differences in Walking Pattern during 6-Min Walk Test between Patients with COPD and Healthy Subjects
Source: PLoS One. 2012 May 18;7(5):e37329. doi: 10.1371/journal.pone.0037329 (PMC3356256; doi:10.1371/journal.pone.0037329)
Supplement: Table S1 — Patients' characteristics for all COPD patients and a subgroup of COPD patients with a 2nd test. (DOCX) [file pone.0037329.s002.docx]

**Table S1: Patients’ characteristics for all COPD patients and a subgroup of COPD patients with a 2^nd^ test.**

|  | **All COPD** | **COPD subgroup** |
| --- | --- | --- |
|  | **(n=79)** | **(n=49)** |
| Men (%) | 59.5 | 63.3 |
| Age (yrs) | 64.3 (8.9) | 63.6 (9.5) |
| Height (m) | 1.67 (0.09) | 1.67 (0.08) |
| Weight (kg) | 69.0 (15.0) | 70.9 (15.5) |
| BMI (kg/m2) | 24.7 (4.5) | 25.4 (4.5) |
| Tiffeneau index (%) | 40.7 (11.9) | 41.7 (12.4) |
| FEV1 (%pred) | 53.5 (18.7) | 56.5 (19.9) |
| RV/TLC ratio (%) | 50.5 (10.0) | 48.9 (10.2) |
| 6MWD (m) | 494 (96) | 511 (79)* |
| 6MWD (% pred) | 77.6 (13.5) | 80.3 (10.7)* |
| Baseline dyspnea (points) | 1.64 (1.22) | 1.47 (1.42)* |
| ∆ Dyspnea (points) | 2.74 (1.97) | 2.73 (1.89)* |
| Baseline fatigue (points) | 1.52 (1.48) | 1.59 (1.23)* |
| ∆ Fatigue (points) | 2.25 (1.76) | 2.33 (1.64)* |
| FFM (kg) | 46.5 (8.8) | 47.3 (8.6) |
| FFMi (kg/m2) | 16.6 (2.2) | 16.8 (2.1) |

*Values are reported for the best 6MWT only.

Abbreviations: BMI: body mass index, FEV1: forced expiratory volume in the first second, RV: residual volume ,TLC: total lung capacity, 6MWD: ,FFM: fat-free mass, FFMi: fat-free mass index
