# Supplementary material for: Differences in Walking Pattern during 6-Min Walk Test between Patients with COPD and Healthy Subjects
Source: PLoS One. 2012 May 18;7(5):e37329. doi: 10.1371/journal.pone.0037329 (PMC3356256; doi:10.1371/journal.pone.0037329)
Supplement: Table S2 — Accelerometer features. (DOCX) [file pone.0037329.s003.docx]

**Table S2: Accelerometer features**

|  | **All COPD** | **COPD best 6MWD** | **COPD worst 6MWD** |
| --- | --- | --- | --- |
|  | **(n=79)** | **(n=49)** | **(n=49)** |
| 6MWD (m) | 494 (96) | 511 (79) | 479 (76)* |
| Walking intensity (counts/min) | 8658 (2971) | 9104 (2867) | 8124 (2335)* |
| Cadence (strides/min) | 57 (6) | 58 (5) | 56 (5)* |
| AC-AP (%) | 79.0 (10.7) | 79.8 (9.7) | 77.9 (10.0) |
| AC-V (%) | 84.2 (10.2) | 85.9 (6.4) | 82.3 (9.7)* |
| AC-ML (%) | 63.2 (14.0) | 64.9 (12.9) | 60.1 (13.3)* |

Value expressed as mean ± standard deviation (SD)

Abbreviations: AC-AP: autocorrelation coefficient in anterior-posterior direction, AC-V: autocorrelation coefficient in vertical direction, AC-ML: autocorrelation coefficient in medio-lateral direction

*: significantly different compared to best 6MWD. (p<0.05)
